# Supplementary material for: Unveiling the Bioactive Potential of the Invasive Jellyfish Phyllorhiza punctata Through Integrative Transcriptomic and Proteomic Analyses
Source: Biomolecules. 2025 Aug 4;15(8):1121. doi: 10.3390/biom15081121 (PMC12383608; doi:10.3390/biom15081121)
Supplement: Supplementary file 1 [file biomolecules-15-01121-s001.zip › Suplementary Material/FileS3/PEPFOLD4/XP_065060742_8629/clusters.html]

 PEP-FOLD clusters


Download archive of all models  here   

|  |  |  |
| --- | --- | --- |
| # | file | sOPEP |
| # cluster 1 - Size: 80 |  |  |
|  | XP\_065060742\_8629-model1 | -15.1649 |
|  | XP\_065060742\_8629-model1.1 | -15.1649 |
|  | XP\_065060742\_8629-model1.2 | -15.1649 |
|  | XP\_065060742\_8629-model1.3 | -15.1649 |
|  | XP\_065060742\_8629-model1.4 | -15.1649 |
|  | XP\_065060742\_8629-model1.5 | -15.1649 |
|  | XP\_065060742\_8629-model1.6 | -15.1649 |
|  | XP\_065060742\_8629-model1.7 | -15.1649 |
|  | XP\_065060742\_8629-model1.8 | -15.1649 |
|  | XP\_065060742\_8629-model1.9 | -15.1649 |
|  | XP\_065060742\_8629-model1.10 | -15.1649 |
|  | XP\_065060742\_8629-model1.11 | -15.1649 |
|  | XP\_065060742\_8629-model1.12 | -15.1649 |
|  | XP\_065060742\_8629-model1.13 | -15.1649 |
|  | XP\_065060742\_8629-model1.14 | -15.1649 |
|  | XP\_065060742\_8629-model1.15 | -15.1649 |
|  | XP\_065060742\_8629-model1.16 | -15.1649 |
|  | XP\_065060742\_8629-model1.17 | -15.1649 |
|  | XP\_065060742\_8629-model1.18 | -15.1649 |
|  | XP\_065060742\_8629-model1.19 | -15.1649 |
|  | XP\_065060742\_8629-model1.20 | -15.1649 |
|  | XP\_065060742\_8629-model1.21 | -15.1649 |
|  | XP\_065060742\_8629-model1.22 | -15.1649 |
|  | XP\_065060742\_8629-model1.23 | -15.1649 |
|  | XP\_065060742\_8629-model1.24 | -15.1649 |
|  | XP\_065060742\_8629-model1.25 | -15.1299 |
|  | XP\_065060742\_8629-model1.26 | -14.9623 |
|  | XP\_065060742\_8629-model1.27 | -14.9623 |
|  | XP\_065060742\_8629-model1.28 | -14.9623 |
|  | XP\_065060742\_8629-model1.29 | -14.9623 |
|  | XP\_065060742\_8629-model1.30 | -14.9623 |
|  | XP\_065060742\_8629-model1.31 | -14.9623 |
|  | XP\_065060742\_8629-model1.32 | -14.807 |
|  | XP\_065060742\_8629-model1.33 | -14.807 |
|  | XP\_065060742\_8629-model1.34 | -14.807 |
|  | XP\_065060742\_8629-model1.35 | -14.807 |
|  | XP\_065060742\_8629-model1.36 | -14.807 |
|  | XP\_065060742\_8629-model1.37 | -14.807 |
|  | XP\_065060742\_8629-model1.38 | -14.807 |
|  | XP\_065060742\_8629-model1.39 | -14.807 |
|  | XP\_065060742\_8629-model1.40 | -14.807 |
|  | XP\_065060742\_8629-model1.41 | -14.807 |
|  | XP\_065060742\_8629-model1.42 | -14.807 |
|  | XP\_065060742\_8629-model1.43 | -14.807 |
|  | XP\_065060742\_8629-model1.44 | -14.807 |
|  | XP\_065060742\_8629-model1.45 | -14.807 |
|  | XP\_065060742\_8629-model1.46 | -14.807 |
|  | XP\_065060742\_8629-model1.47 | -14.807 |
|  | XP\_065060742\_8629-model1.48 | -14.807 |
|  | XP\_065060742\_8629-model1.49 | -14.6972 |
|  | XP\_065060742\_8629-model1.50 | -14.6972 |
|  | XP\_065060742\_8629-model1.51 | -14.6972 |
|  | XP\_065060742\_8629-model1.52 | -14.6972 |
|  | XP\_065060742\_8629-model1.53 | -14.6972 |
|  | XP\_065060742\_8629-model1.54 | -14.6972 |
|  | XP\_065060742\_8629-model1.55 | -14.683 |
|  | XP\_065060742\_8629-model1.56 | -14.683 |
|  | XP\_065060742\_8629-model1.57 | -14.683 |
|  | XP\_065060742\_8629-model1.58 | -14.683 |
|  | XP\_065060742\_8629-model1.59 | -14.683 |
|  | XP\_065060742\_8629-model1.60 | -14.683 |
|  | XP\_065060742\_8629-model1.61 | -14.683 |
|  | XP\_065060742\_8629-model1.62 | -14.683 |
|  | XP\_065060742\_8629-model1.63 | -14.683 |
|  | XP\_065060742\_8629-model1.64 | -14.683 |
|  | XP\_065060742\_8629-model1.65 | -14.6805 |
|  | XP\_065060742\_8629-model1.66 | -14.4928 |
|  | XP\_065060742\_8629-model1.67 | -14.4928 |
|  | XP\_065060742\_8629-model1.68 | -14.4011 |
|  | XP\_065060742\_8629-model1.69 | -14.4011 |
|  | XP\_065060742\_8629-model1.70 | -14.4011 |
|  | XP\_065060742\_8629-model1.71 | -14.4011 |
|  | XP\_065060742\_8629-model1.72 | -14.2626 |
|  | XP\_065060742\_8629-model1.73 | -14.2288 |
|  | XP\_065060742\_8629-model1.74 | -14.2288 |
|  | XP\_065060742\_8629-model1.75 | -14.2288 |
|  | XP\_065060742\_8629-model1.76 | -14.2288 |
|  | XP\_065060742\_8629-model1.77 | -14.2288 |
|  | XP\_065060742\_8629-model1.78 | -14.2288 |
|  | XP\_065060742\_8629-model1.79 | -14.2098 |
| # cluster 2 - Size: 15 |  |  |
|  | XP\_065060742\_8629-model2 | -9.35501 |
|  | XP\_065060742\_8629-model2.1 | -9.1608 |
|  | XP\_065060742\_8629-model2.2 | -9.06271 |
|  | XP\_065060742\_8629-model2.3 | -8.94413 |
|  | XP\_065060742\_8629-model2.4 | -8.94413 |
|  | XP\_065060742\_8629-model2.5 | -8.85185 |
|  | XP\_065060742\_8629-model2.6 | -8.62096 |
|  | XP\_065060742\_8629-model2.7 | -8.58231 |
|  | XP\_065060742\_8629-model2.8 | -8.562 |
|  | XP\_065060742\_8629-model2.9 | -8.3798 |
|  | XP\_065060742\_8629-model2.10 | -8.15905 |
|  | XP\_065060742\_8629-model2.11 | -7.99696 |
|  | XP\_065060742\_8629-model2.12 | -7.94445 |
|  | XP\_065060742\_8629-model2.13 | -7.17344 |
|  | XP\_065060742\_8629-model2.14 | -6.6707 |
| # cluster 3 - Size: 2 |  |  |
|  | XP\_065060742\_8629-model3 | -8.88973 |
|  | XP\_065060742\_8629-model3.1 | -6.68073 |
| # cluster 4 - Size: 1 |  |  |
|  | XP\_065060742\_8629-model4 | -7.96292 |
| # cluster 5 - Size: 2 |  |  |
|  | XP\_065060742\_8629-model5 | -7.58202 |
|  | XP\_065060742\_8629-model5.1 | -7.21453 |
|  |  |  |
